# Association of Immunological Cell Profiles with Specific Clinical Phenotypes of Scleroderma Disease

**DOI:** 10.1155/2014/148293

**Published:** 2014-04-10

**Authors:** José Manuel López-Cacho, Soledad Gallardo, Manuel Posada, Miriam Aguerri, David Calzada, Teodoro Mayayo, María Luisa González-Rodríguez, Antonio María Rabasco, Carlos Lahoz, Blanca Cárdaba

**Affiliations:** ^1^Department of Immunology, IIS-Jiménez Díaz Foundation, Reyes Católicos Avenue 2, 28040 Madrid, Spain; ^2^Institute of Rare Diseases Research, Carlos III Institute of Health, EuroBioBank, and CIBERER, 28029 Madrid, Spain; ^3^Sani-Red S.L, Barcelona Scientific Park, 08013 Barcelona, Spain; ^4^Department of Pharmaceutical Technology, Faculty of Pharmacy, University of Seville, 41012 Seville, Spain; ^5^CIBER of Respiratory Diseases (CIBERES), 28029 Madrid, Spain

## Abstract

This study aimed to search the correlation among immunological profiles and clinical phenotypes of scleroderma in well-characterized groups of scleroderma patients, comparing forty-nine scleroderma patients stratified according to specific clinical phenotypes with forty-nine healthy controls. Five immunological cell subpopulations (B, CD4^+^ and CD8^+^ T-cells, NK, and monocytes) and their respective stages of apoptosis and activation were analyzed by flow cytometry, in samples of peripheral blood mononuclear cells (PBMCs). Analyses of results were stratified according to disease stage, time since the diagnosis, and visceral damage (pulmonary fibrosis, pulmonary hypertension, and cardiac affliction) and by time of treatment with corticosteroids. An increase in the percentages of monocytes and a decrease in the B cells were mainly related to the disease progression. A general apoptosis decrease was found in all phenotypes studied, except in localized scleroderma. An increase of B and NK cells activation was found in patients diagnosed more than 10 years ago. Specific cell populations like monocytes, NK, and B cells were associated with the type of affected organ. This study shows how, in a heterogeneous disease, proper patient's stratification according to clinical phenotypes allows finding specific cellular profiles. Our data may lead to improvements in the knowledge of prognosis factors and to aid in the analysis of future specific therapies.

## 1. Introduction

Scleroderma is a rare autoimmune disease of unknown etiology which affects thousands of people around the world. Its prevalence is estimated to be between 15 and 35 cases per 100000 inhabitants [1–3]. Its first symptoms can be seen around the third and fourth decade of the life but, in some cases, symptoms can exist for several years without a correct diagnosis. Scleroderma is three times more common in women than men. The disease is not linked in any consistent way to race, season, geography, occupation, or socioeconomic status. Environmental etiologies are nonetheless possible [1].

Scleroderma is a complex autoimmune disease characterized by fibrosis in all the organs, although its name is derived from the fibrosis of skin caused by the disease. Damage in the endothelium seems to be the initial lesion responsible for the cascade of events that results in the disease [4, 5] leading three main types of alterations: vascular occlusion, immune system alterations, and connective tissue proliferation.

Fibrosis of the internal organs leads to respiratory problems, dysphagia, bowel alterations, and kidney and cardiac dysfunctions; these complications lead to marked disability, loss of quality of life, and high rates of mortality [1]. There are two main types of scleroderma, localized and systemic. Localized scleroderma affects mainly the skin, while systemic scleroderma may affect many parts of the body. Localized scleroderma (LSc) can be classified as morphea, linear, or linear “en coup de sabre.” Systemic scleroderma (SSc) can be divided into three major subtypes, limited cutaneous systemic sclerosis (lcSSc), diffuse cutaneous systemic sclerosis (dcSSc) (sclerosis of proximal extremities, trunk, and face), and systemic sclerosis sine scleroderma (organ fibrosis only; no skin thickening).

Differences have been described in immunological cell subpopulations in patients with different types of complications and visceral involvement [6–8]. The results of studies in scleroderma patients which have investigated the number and the percentages of lymphocytes [9–12], their activation [13, 14], and apoptosis states [14, 15] have shown some discrepancies. Reasons for these discrepancies are not clearly apparent but could be related to differences in subtype, stage, and activity of the disease, demographic characteristics of the patients, and methodological issues in obtaining and analyzing patient samples [15]. To clarify these discrepancies, we have studied the relationship between clinical scleroderma phenotypes and the different cellular subpopulations in well-characterized groups of scleroderma patients, comparing their results with healthy controls and stratifying them according to scleroderma subtypes, time since the diagnosis of the disease, and presence of complications (pulmonary fibrosis, pulmonary hypertension, and cardiac affliction) and by time with corticosteroids treatment. Clinical characteristics like gastroesophageal reflux, digital ulcers, joint involvement, scleroderma renal crisis, or Rodnan total skin [16] score were not considered phenotypic strata in this study.

## 2. Materials and Methods

### 2.1. Subjects

Scleroderma patients and controls were ascertained from Spanish Association of Scleroderma (AEE), with the collaboration of the Institute of Rare Diseases Research (IIER), Instituto de Salud Carlos III (ISCIII), and the Spanish Federation of Rare Diseases (FEDER). As all scleroderma patients were prevalent cases, we validated their diagnosis checking that they met the classification criteria for SSc established by the ARA in 1980 [17] instead of the new one established in 2013 by the ACR/EULAR [18].

The identification of the different types of scleroderma as well as the organ damages was based on clinical features extracted from the clinical records which were made by rheumatologists and/or internal medicines specialists at hospitals of Spain. Time elapsed since the onset of the first symptoms to the diagnosis of the disease was estimated through telephone surveys, with the cases who accepted to participate in this study. Clinical medical records were revised to confirm some dates, when necessary criteria for pulmonary fibrosis, heart lesions, and pulmonary hypertension were obtained from echocardiography and/or pulmonary function tests stated in clinical records.

Controls were selected according to the age and gender of each patient recruited who was responsible for providing his/her own controls among his/her friends and/or neighborhoods. Controls demonstrated no clinical findings suggestive of infections, allergic manifestations, or immunological disorders.

Forty-nine scleroderma patients (43 females and 6 males) and 49 healthy subjects (26 females and 23 males) were included in the study. Although there was a higher presence of females in scleroderma patients than in control group (87.7% versus 53.06%), the analysis according to sex did not show any statistically significant association with cellular population studied. The mean age ± standard deviation in the total group of patients was 48.0 ± 10.9 years and 48.4 ± 13.1 years for controls (Table 1). During the assays, thirteen patients were receiving corticosteroid treatment (11 with prednisone, 1 with methylprednisolone, and 1 with deflazacort), 4 patients were undergoing treatment with other immunosuppressive drugs, and 11 patients were treated with a combination of an immunosuppressive with a corticosteroid. None of the patients took the medication in the 12 hours before the samples were collected. All participants signed an informed consent in which they agreed to participate in the project. The project was also approved by the Ethics Committee of both participating institutions (Instituto de Salud Carlos III and IIS-Fundación Jiménez Díaz).

### 2.2. Isolation of Peripheral Blood Mononuclear Cells (PBMCs)

PBMCs from donors were isolated from venous blood by density gradient sedimentation on Lymphoprep (Comercial Rafer, Zaragoza, Spain) following the manufacturer's instructions and resuspended in freezing medium containing 90% FBS (Lonza, Verviers, Belgium) and 10% DMSO (Sigma-Aldrich, St. Louis, USA). The cells were aliquoted into cryogenic vials and stored in liquid nitrogen until use. PBMCs were thawed in RPMI 1640 supplemented with 5% FBS inactivated, 1% L-glutamine, 1% peni-streptomycin, and 1 mM sodium pyruvate (Flow Laboratories, Irvine, UK) and after two washed, the PBMCs were resuspended in saline buffer and stained according recommendations of the specific commercial antibody. Quantification and viability were determined by trypan blue exclusion. Before doing apoptosis and activation analysis, controls with freeze PBMCs samples were carried out for testing quality of assays. Samples included for analysis had a viability of at least 90%. All biological samples were collected from the Institute of Rare Diseases Research Biobank, belonging to European Rare Diseases Biobank-EuroBioBank.

### 2.3. Immunophenotyping

**Figure 1:**
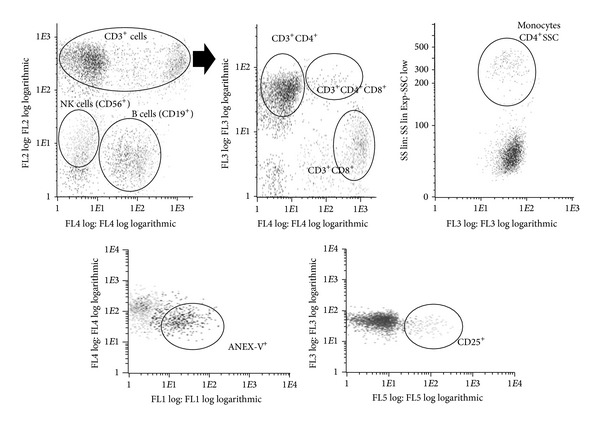
Characterization of cellular subpopulations analyzed by flow cytometry. FL1: Annexin-V^+^ cells; FL2: CD3^+^ or/and CD56^+^ cells; FL3: CD4^+^ cells; FL4: CD19^+^ or CD8^+^ cells; FL5: CD25^+^ cells; SSC: side-scatter detector.

Cellular phenotype was analyzed by flow cytometry using a single PBMCs sample labeled with a five-color combination of monoclonal antibodies for Annexin-V-FITC (FL-1) (Ref: ANXVKF-100T, Inmunostep, Salamanca, Spain), CD3-PE (Ref: CYT-3PE5) and CD56-PE conjugated (FL-2) (Ref: CYT-56PE) (Vitro-Cytognos, Salamanca, Spain), CD4-ECD (FL-3) (Ref: 6604727, Beckman Coulter, Izasa, Barcelona, Spain), CD8-PC5 (Ref: CYT-8C2) and CD19-PC5 conjugated (FL-4) (Ref: CYT-19C2) (Vitro-Cytognos, Salamanca, Spain), and CD25-PC7 (FL-5) conjugated (Ref: PN A52882, Beckman Coulter, Izasa, Barcelona, Spain) by using Cytomics FC500 and CXP software (Beckman Coulter, Brea, USA). Unlike other studies, the different signal intensities of this combination of fluorochromes allowed us to analyze several clusters of differentiation at the same time using only one determination (Figure 1). At least 5,000 events were acquired in a list mode and all the data were analyzed by cell quest software (CXP Beckman Coulter).

Five-color flow cytometry analysis was performed as follows. Briefly, 2 · 10^5^ PBMCs were labeled with 20 *μ*L of each monoclonal antibody (CD3-PE, CD56-PE, CD4-ECD, CD8-PC5, CD19-PC5, and CD25-PC7 conjugated) and placed in an ice-water bath for 20 minutes.

After washing with PBS, 20 *μ*L of Annexin-V-FITC was delivered and the tubes were placed in an ice-water bath for 15 minutes in darkness. Then, the cells were resuspended in 200 *μ*L of binding buffer (PBS) for immediate flow cytometry analysis.

Live PBMCs were electronically gated by forward- and right-angle scatters; different types of cells were then gated and the percentage and intensity of expression were evaluated. When the gated cells were uncertain or very few, the data were excluded. Negative controls included a human isotype-matched nonrelevant immunoglobulin: Mouse IgG2a-PE (CYT-IC006PE, Cytognos) and IgG2b-PE (CYT-IC007PE, Cytognos), IgG1-PECy5 Mouse Isotypic Control (CYT-IC005C, Cytognos), Mouse IgG2a- PC7 (PN A12692, Beckman Coulter), and Mouse IgG1-ECD Isotypic Control (A07797, Beckman Coulter). Figure 1 (upper panel) summarizes the selection of cell populations studied: percentages of T lymphocytes CD3^+^CD4^+^ and CD3^+^CD8^+^ were evaluated for the total number of CD3^+^ cells. Percentages of B cells (CD3^−^CD19^+^), NK cells (CD3^−^CD56^+^), and monocytes were evaluated regarding total number of cells. Monocytes were gated based on CD3^−^CD4^+^ expression and high side-scatter (SSC).

After the determination of cellular subpopulations, the percentages of apoptosis (Annexin-V^+^) and activation (CD25^+^ expression) of each subpopulation were evaluated regarding their respective overall number of cells (lower panel of Figure 1).

### 2.4. Statistical Analysis

Global analysis was performed by Kruskal-Wallis test and comparisons between groups were performed using a nonparametric two-tailed Mann-Whitney test for independent variables. A *P* value of <0.05 was used. We used the R program for the statistical analysis and the box-plots design.

## 3. Results

Table 2 shows the medians of the percentages of each type of cellular subpopulations studied, as well as their activation or apoptosis stages, grouped by clinical phenotype.

Significant differences were detected in scleroderma patients when we compare their results with the control group. We found a decrease in the percentage of B lymphocytes and higher values of monocytes. We did not find any difference in activation states, although for apoptosis values we observed a decrease in all the subsets of cells compared to controls.

**Figure 2:**
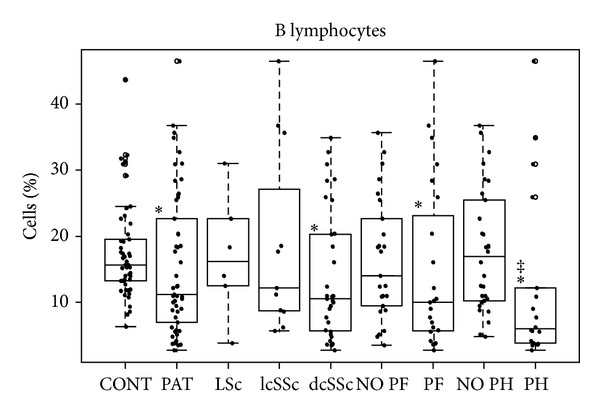
Box-plot of B lymphocytes.*Statistically significant relative to controls. ^‡^Statistically significant relative to patients without pulmonary hypertension.

**Figure 3:**
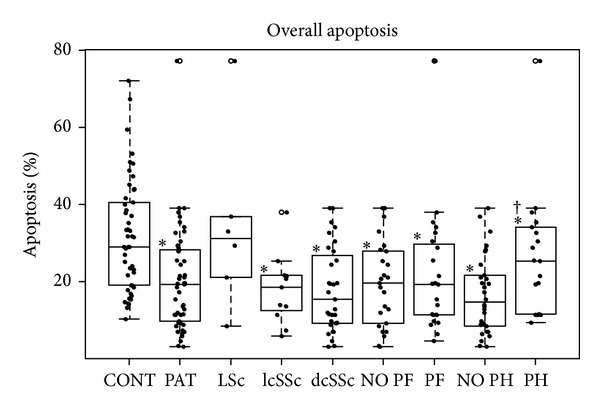
Box-plot of overall apoptosis regarding scleroderma type and presence of PH and PF. *Statistically significant relative to controls. ^†^Statistically significant relative to patients without pulmonary hypertension.

According to scleroderma phenotypes, we found a progressive reduction in the percentages of B lymphocytes in the most severe phenotypes of the disease (Figure 2); this decrease in the diffuse systemic variant (*P* < 0.01) compared to controls was statistically significant. A statistically significant increase in monocytes was shown relative to controls (3.35%) only in patients affected by systemic sclerodermas (7.41% in limited form and 7.91% in diffuse form). No difference in cell activation was found among scleroderma phenotypes. As for overall apoptosis, we found a decrease in systemic but not in localized sclerodermas (Figure 3). Apoptosis values by cellular population (Table 2) were decreased in several cellular subsets in systemic sclerodermas. The reduction was in all the subsets analyzed in the diffuse form and B cells and in monocytes in the limited form. Localized sclerodermas showed an apoptotic decrease in monocytes.

**Figure 4:**
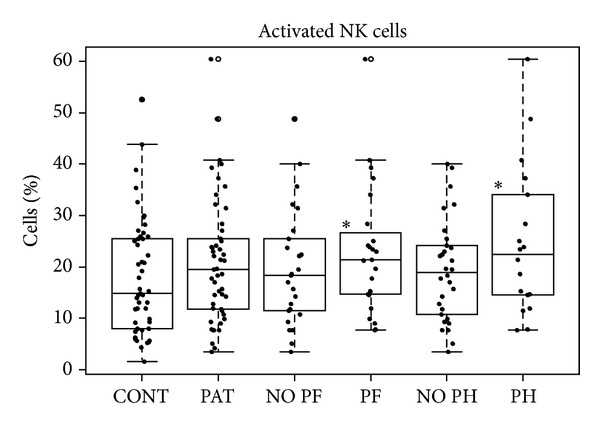
Box-plot of activated NK cells. *Statistically significant relative to controls.

In patients with pulmonary fibrosis (PF), we found a reduction in the percentages of B lymphocytes (Figure 2) and an increase of activated NK cells (Figure 4). Patients with and without fibrosis showed increased monocyte percentages (Table 2) and similar apoptotic profiles. We obtained reduced percentages in all cell subpopulations except in CD4 T-cells in patients with PF.

In the group of patients with pulmonary hypertension (PH), we found the lowest percentages of B cells (Figure 2). This group was the only one without an increase of monocytes (Table 2). We also found significant increases of activated B, NK, and CD8^+^ cells. The increase of CD8^+^ cells is altered like in pulmonary fibrosis. Regarding apoptosis, we found decreases in all the subpopulations of patients without pulmonary hypertension when comparing these subpopulations to controls. In addition, we found statistically significant differences when comparing absence and presence of PH, with lower apoptosis found in the absence of pulmonary hypertension (Table 2).

We found few significant differences in patients affected by cardiac affliction (CA) probably due to the low number of patients tested (*n* = 7); we only found an increase of monocytes (Table 2).

Regarding time since the diagnosis of the disease, we only found an increase of monocytes in patients diagnosed from 4 to 10 years (Table 2). Patients diagnosed more than 10 years previously showed an increase in the percentages of activated B and NK cells; these results were very similar to those obtained in patients diagnosed from 0 to 3 years (Table 2). We also found the lowest percentages of apoptosis in the group of patients diagnosed from 4 to 10 years ago (Table 2), but a progressive recovery is produced with time, except for B cells and monocytes.

Regarding to the treatment time with corticosteroids, thetreatment decreases the percentages of B cells and, in patients treated during more than 3 years, a reduction of the percentages of monocytes and their activation state are produced (Table 2). In this group we also obtained the smallest percentages of apoptosis (Table 2). In addition, the group of patients diagnosed from zero to three years had higher percentages of activated NK cells.

## 4. Discussion

There have been conflicting results concerning changes in the immune cell subpopulations involved in scleroderma [11, 19–21] due to the heterogeneity of the disease and differences in study methods. In order to shed light on this issue, we have studied a well-defined clinical population, analyzing both the cell subpopulations and their activation and apoptosis stages and also correlating the data with different clinical phenotypes.

We obtained normal values of T lymphocytes, as has been described by other authors [11, 20–22]. However, we found a reduction of B-cells in the worse-prognosis situations of the disease but higher percentages of monocytes in almost all SSc subsets. These results can be also correlated with the conditions of visceral involvement that triggers the worst prognosis (Figure 2 and Table 2).

The monocyte fraction of peripheral blood contains precursors to potential regulators of fibrosis [23] such as fibrocytes and macrophages [18, 24, 25]. Several studies obtained functional abnormalities and increased numbers of these cells in scleroderma patients [23, 26–29]. Our results are consistent with many data that indicate the association between elevated levels of peripheral blood fibrocytes and monocytes and diverse forms of organ remodeling such as renal fibrosis, cirrhosis, or different types of pulmonary fibrosis [29]. An abnormal remodeling in scleroderma is usually more common in systemic sclerodermas [30]. However, our monocytes results must be considered with caution because monocytes were not detected directly through a CD14 marker and, instead, were selected indirectly, as CD3^−^CD4^+^, and some monocytes cannot express CD4.

The action of B cells in scleroderma could be related to their functions in the regulation of the immune response [31] and antibody production and abnormalities in them can induce or develop autoimmune diseases like scleroderma.

Analyzing the activation states, we obtained a higher overall activation in patients in whom the time elapsed since diagnosis was greater or in patients with visceral involvement (especially in B and NK cells). Some authors determine that survival rates of scleroderma patients begin their decline in the period of time of four to ten years since they go to the hospital [32, 33]. As detected in our study, the development of serious visceral involvement portends early mortality in patients with scleroderma [32, 34, 35] and is concomitant with elevated activation states of immune cells [28, 36, 37]. The role played by NK cells in autoimmune diseases is both a disease-controlling and a disease-promoting role and NK cells may be implicated in the onset, maintenance, or progression of these diseases [38], thus suggesting their involvement in the scleroderma [39].

Activation markers like CD25 play a role in cell contact interactions between T-cells and other cells, including fibroblasts. However, the analysis of other lymphocytes activation markers as CD69 or MHC Class II could be interesting, especially for determining monocyte activation. Inclusion of cytokine production from these cell populations could also enhance information about their states of activation.

Apoptosis values may be difficult to evaluate due to the method for conserving the cells. In this study, as frozen cells were used, the freezing effect was checked and was homogenous in all samples. Annexin V staining precedes the loss of membrane integrity which accompanies the later stages of cell death resulting from either apoptotic or necrotic processes, for that was selected as cellular death marker.

We found a decrease in different subsets in the disease, possibly because of apoptosis resistance in the cells of scleroderma patients. Apoptosis resistance is the primary mechanism leading to the development of fibrotic lesions and could be one of the reasons why some types of sclerodermas have more visceral fibrosis and worse prognosis, especially in the case of diffuse forms, where the apoptosis rates are the lowest. Some authors [40] have described that, during later phases of systemic sclerosis, inflammatory events become less intense and the immune response acts as a low-grade amplifier of fibrogenesis and microangiopathy, presenting a significant therapeutic challenge. This could explain the differences that we have found between the patients analyzed according to time since diagnosis: a diminution in the beginning of the disease, which could coincide with the endothelial damage; after four to ten years, the disease could coincide with the development of fibrosis in the internal organs, where we have obtained apoptosis diminutions; and, finally, until about ten years following diagnosis, the apoptosis tends to increase and the fibrosis tends to slow down.

In patients diagnosed more than ten years previously, the reduction in the apoptosis of B cells and monocytes is parallel to an increase in the overall activation, especially in the activation of B and NK cells. This coincides with decreases in the survival rates of the scleroderma patients [32, 33] and organ affliction tends to appear in the late-onset stages of the disease [30]. The detected alterations in these subpopulations could have some influence on survival rates and could be useful in the prognosis of the disease.

The usual treatment of scleroderma—especially of organ fibrosis—[30] is based on immunosuppressive drugs and corticosteroids that increase the apoptosis of the inflammatory cells [41]. In our results, the treatment seems to increase the apoptosis in the group treated with corticosteroids from zero to three years but, in patients with more than three years treated, the situation reverts to diminished percentages. This could be one of the reasons that make treatment with corticosteroids inefficient in patients who have had the disease for many years. In addition to this, two things must be taken into account: both kinds of drugs can interfere with the action of B cells, although they usually do not reduce their numbers and corticosteroids produce an inhibitory effect in NK cells [42] but these cells have a lower sensitivity to these kinds of drugs [43]. These data coincide with our results where more time is needed to reduce their activation. However, corticosteroids are useful in monocytes, producing a reduction in the patients with more time with the disease.

## 5. Conclusions

In conclusion, an overall decrease in apoptosis and in the numbers of B cells together with an increase of monocytes and activated B and NK cells is directly correlated with the severity of the disease. These results suggest that these cells contribute to the immunological abnormalities observed in scleroderma and could announce the appearance of complications or a worsening in the disease.

## Figures and Tables

**Table 1:** Participant distribution by subsets.

Comparison	Group	Patients
Controls	CONT	49

Patients	PAT	49

Type of scleroderma	LSc	6
	dcSSc	32
	lcSSc	11

Time since the diagnosis of the disease (years)	0–3	13
	4–10	15
	>11	21

Treatment with corticoids (years)	No	21
	0–3	17
	>3	11

Pulmonary fibrosis	No	26
	Yes	23

Cardiac affliction	No	42
	Yes	7

Pulmonary hypertension	No	31
	Yes	18

**Table 2:** Cell subpopulation percentages (medians) stratified by subsets.

Cell population	Overall		Type			Pulmonary fibrosis		Pulmonary hypertension		Cardiac affliction		Time since the diagnosis of the disease (years)			Corticosteroids treatment (years)		
	Controls *n* = 49	Patients *n* = 49	LSc *n* = 6	lcSSc *n* = 11	dcSSc *n* = 32	No *n* = 26	Yes *n* = 23	No *n* = 31	Yes *n* = 18	No *n* = 42	Yes *n* = 7	0–3 *n* = 13	4–10 *n* = 15	>10 *n* = 21	No *n* = 21	0 to 3 *n* = 17	>3 *n* = 11
General																	
CD4^+^ T-cells	65.04	62.46	62.36	66.17	63.84	62.36	63.84	65.66	62.19	62.46	68.35	64.16	63.84	61.91	62.31	62.46	65.46
CD8^+^ T-cells	29.86	27.16	28.45	25.77	25.14	27.66	25.65	26.15	27.5	27.66	25.14	28.81	24.44	29.13	25.26	29.23	25.14
B cells	15.62	**11.14**	16.19	12.13	**10.56**	14.02	**9.97**	16.87	**5.98** ^‡^	12.39	6.19	12.51	12.13	11.01	18.50	**10.56**	**9.97**
NK cells	11.09	11.73	10.09	9.74	11.77	9.89	12.4	10.51	12.7	11.65	12.34	9.43	11.65	12.34	14.81	9.89	10.39
Monocytes	3.35	**7.41**	4.65	**7.41**	**7.91**	**7.41**	**7.25**	**7.66**	3.78	**7.25**	**12.81**	6.87	**7.53**	4.43	**6.87**	7.53	4.43
Activation																	
Overall	7.32	8.07	9.37	8.68	6.46	6.46	8.63	6.87	8.67	8.20	6.91	8.38	5.14	9.52*	8.67	8.68	6.46
CD4^+^ T-cells	5.05	6.88	7.81	8.47	5.78	5.78	7.21	5.54	7.08	6.69	7.99	8.17	4.67	7.44	5.23	7.69	6.69
CD8^+^ T-cells	4.34	5.66	7.00	5.29	4.49	5.29	8.25	5.08	**8.28**	6.09	4.17	6.61	3.87	7.09	7.56	6.90	4.17
B cells	9.79	11.61	13.11	5.41	11.98	11.87	11.48	11.33	**15.69** ^†^	11.87	10.00	12.19	9.03	**13.48**	8.54	13.48	14.71
NK cells	14.93	19.57	24.57	22.41	15.68	18.41	**21.39**	18.99	**22.44**	19.57	21.30	**23.27**	11.76	**23.48**	17.05	**23.09**	17.71
Monocytes	70.80	70.10	81.75	66.67	61.90	77.36	59.42	74.88	61.52	74.75	58.60	62.93	84.68	61.90	84.45	70.66	**50.00**
Apoptosis																	
Overall	29.00	**19.16**	31.23	**18.51**	**15.41**	**19.67**	**19.16**	**14.67**	**25.32** ^†^	**19.16**	19.61	**16.77**	**15.41**	**25.22**	**20.21**	**19.61**	**12.88**
CD4^+^ T-cells	22.56	**17.48**	22.61	13.97	**11.05**	**17.66**	17.48	**11.10**	**21.88** ^†^	**17.48**	21.36	13.23	**11.05**	22.79	18.44	18.22	11.05
CD8^+^ T-cells	21.42	**16.75**	28.57	21.05	**10.63**	16.67	21.05	**10.49**	24.8	**16.75**	22.00	21.07	**10.35**	27.08	16.71	21.69	**9.84**
B cells	30.97	**19.42**	38.59	**15.52**	**18.92**	**19.12**	**20.92**	**15.26**	**28.13** ^†^	**20.92**	**18.92**	**18.85**	**19.12**	**22.35**	**20.89**	24.68	**18.92**
NK cells	45.68	**35.43**	68.02	37.93	**30.77**	37.93	32.67	**33.10**	45.32	**36.69**	32.67	37.31	**27.99**	40.81	**31.18**	47.54	**33.33**
Monocytes	80.00	**62.90**	**59.67**	**65.97**	**62.90**	**58.58**	**66.67**	**58.32**	67.48	**62.90**	62.04	**57.44**	**62.04**	**66.26**	**62.93**	**66.41**	**55.88**

Bold letters indicate statistically significant results relative to controls. ^‡^Statistically significant compared to controls and without pulmonary hypertension. ^†^Statistically significant relative to patients without pulmonary hypertension. *Indicate those data statistically significant compared to four and ten years from the diagnosis of the disease.
